# Monitoring and simulation of the fuel irradiation behavior in nuclear reactors based on phononic crystal structure

**DOI:** 10.1038/s41598-023-39298-w

**Published:** 2023-07-29

**Authors:** Fatma A. Sayed, Hussein A. Elsayed, M. F. Eissa, Arafa H. Aly, Ahmed Mehaney

**Affiliations:** TH-PPM Group, Physics Department, Faculty of Sciences, Beni-Suef University, Beni Suef, 62514 Egypt

**Keywords:** Materials science, Mathematics and computing, Optics and photonics, Physics

## Abstract

We have presented in the current work a novel idea for simulating the irradiation behaviors of the nuclear fuel pellets in nuclear reactors by using a one-dimensional defective phononic crystal (1D-DPnC) design was presented. The transmission spectra of the incident mechanical waves were considered basic data for expressing the characteristics of different nuclear fuel-pellets. Herein, the density, sound speed, and Young’s modulus of the fuel-pellets represent the key parameters that are influenced by the irradiation behaviors of these pallets. Mixed plutonium–uranium oxide (MOX) nuclear fuel is considered the main fuel in the present study. In addition, a comparison is performed for this fuel with other types of nuclear fuels. Moreover, the mechanical properties of these MOX-pellets are dependent on the porosity, the ratio of oxygen-to-metal (O/M), and the plutonium (Pu-content). The theoretical treatments depend on the transfers matrix method to compute the transmission spectra through the 1D-DPnC. The numerical findings provided that the MOX-pellet has the highest performance compared to other fuel pellets and with sensitivity equal to 59.388 × 10^3^ Hz s/m. It was also reported that the effects of the percentage of the O/M and Pu- content in MOX had a minor effect in a comparison with the impact of porosity. The theoretical simulation agreed extremely with the experimental data reported for these nuclear fuels. Because of the close relationship between sound speed and density, this sensor can be utilized to monitor the porosity, O/M, Pu-content, and density of fuel-pellets as a quick and non-destructive evaluation technique in a nuclear fuel fabrication laboratory. This article has proven theoretically that MOX fuel produced from nuclear waste of uranium dioxide and plutonium dioxide gives excellent results compared to other types of nuclear fuels, and this agrees with experimental researches. Thus, it may contribute in preserving the environment from nuclear waste, and this can be considered a novel kind of purification of environmental pollution treatment.

## Introduction

Irradiated fuel is exposed to extreme conditions such as a strong radiation field and huge temperature gradient, which results in a complex phase relation and microstructure. The amount of fission product (FP) elements that are created and accumulated during irradiation also has a significant impact on the thermal and mechanical properties of the irradiated fuel^1^. So, when constructing nuclear fuels and simulating their irradiation behaviors in a reactor, the mechanical and thermal properties of these nuclear fuels are essential factors^2–6^. In addition, the fuel materials used in reactors in the form of pellets face degradation in their mechanical properties during the irradiation process^7–10^. As a result, characterizing the mechanical behavior of the radioactive fuel pellets is important to evaluate the integrity and life of the fuel rod. This characterization often involves the use of micro-indentation and micro-acoustic methods^7–9^.

The main parameters of the mechanical properties of any nuclear fuel pellets and any material are represented by Young’s modulus (Y), Shear modulus (S), Bulk modulus (B), Poisson’s ratio (P), etc.^2,9,11,12^. By measuring the sound velocity of the longitudinal and transverse waves within the pellets, the mechanical and thermal properties of nuclear fuel pellets were measured^13^. According to experimental research, these properties are dependent on many parameters of nuclear fuel pellets such as porosity, (O/M), temperature, and Pu- content^3,9,11,12,14,15^. Ref.^12^ used dynamic simulations to study the primary radiation damage in solid solutions of (U_1y_, Pu_y_)O_2_ at varied temperatures and plutonium contents. Ref.^9^ approved a novel method based on longitudinal ultrasonic velocity and porosity for estimating the elastic properties of nuclear fuel materials. Novel correlations have been established between the elastic moduli of these materials. Ref.^8^ used local acoustic measurements made by acoustic microscopy and micro echography, respectively, to demonstrate the laws of variation of the Rayleigh velocity and the longitudinal velocity of the acoustic waves as a function of the porosity in uranium oxide UO_2_. Ref.^11^ have studied the effect of the porosity, (O/M), and (Pu-content) on the sound speed of the longitudinal and transverse waves and mechanical properties of the MOX-pellets. In addition, they have described each parameter that affects the sound speed and the mechanical properties by linear function equations.

Today's biosensor technology has produced clever solutions and different methods for detecting numerous diseases, pharmaceutical liquids, petrochemicals, and even environmental pollutants at the lowest levels^16–20^. Although electronic sensors make up the majority of sensors in use nowadays, new applications have been developed to record mechanical or optical signals. High sensitivity and excellent performance are important attributes that define optical and acoustic sensors^21^. However, in addition to being difficult to fabricate, optical sensors have a disadvantage due to the size and kind of materials utilized^22^. More recently, new sensors based on the variation in acoustic properties of materials could solve these issues. Unquestionably, phononic crystals (PnCs)-based acoustic and elastic sensors might reduce viscosity losses and acoustic/elastic energy dissipations to the limit that they could be produced on a microscale^23^. PnC is a periodic geometry, created from the expression of lattice vibration, which refers to phonon. In addition, PnCs are artificial composites that are created by periodically altering two or more materials in one-dimension (1D), two-dimension (2D), or three-dimension (3D) structures. The most notable characteristic of these structures is the phononic bandgap (PBG), a region in which elastic/acoustic waves cannot propagate. PnCs with a defect layer have drawn a lot of interest since a very thin defect mode with high transmittance^24–29^. The acoustic wave at a particular energy can pass through the PBG as a result of the defect mode^30^. PnCs with a defect layer have known as PnC sensors, and they are used to monitor changes in a material's mechanical properties (mass density or sound speed, etc.). As a result, various sensing applications have been made using 1D, 2D, and 3D PnC sensors^30–33^. Based on all this information, the 1DPnC will be used here with a defect layer filled with different nuclear fuels to simulate the mechanical properties of these nuclear fuels.

In this paper, we shall be dealing first with different nuclear fuels, and design a model based on the 1D-DPnC to simulate the irradiation behaviors of these nuclear fuels. Where the resonance frequency through the (PBG) is the cornerstone for simulating the behavior of these nuclear fuels. After that, the MOX fuel will be the main point of the study and simulate the behavior of its mechanical properties at different percentages from the porosity, O/M, and Pu-content. Finally, the performance parameters of our sensor will be studied for all the simulated behaviors.

## Model design and basic equations

**Figure 1:**
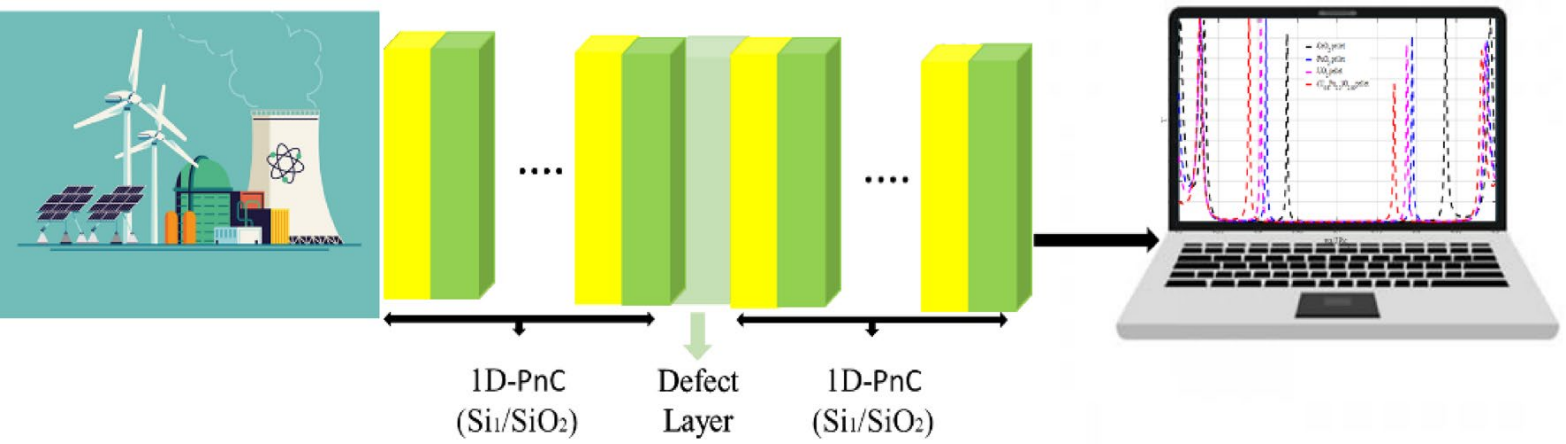
Schematic diagram of the 1D- defective PnC.

The suggested 1D-PnC sensor is presented schematically in Fig. 1. The sensor construction consists of a defect layer filled with different nuclear fuel pellets presented between the two multilayer structures of PnCs. The left and right PnCs consist of repeated unit cells, which consist of two different materials configured as (Si/SiO_2_)^9^. As a result, the proposed structure is constructed using the configuration [(Si/SiO_2_)^9^(D-layer) (Si/SiO_2_)^9^]. The thicknesses of Si, SiO_2,_ and the defect layer are indicated by ${d}_{1}$, ${d}_{2}$, and ${d}_{d}$_,_ and the lattice constant (the thickness of the unit cell) is given by ${a}_{i}={d}_{1}+{d}_{2}$. The materials of our structure are indicated by the subscript $j = 1,{ }2,\;{\text{and}}\;{\text{d}}$. The mechanical properties of our materials are represented in the density, the longitudinal, and the transverse velocities donated by ${\rho }_{j}$, ${C}_{Lj}$ and ${C}_{Tj}$, respectively.

The transfer matrix method (TMM), the plane wave expansion (PWE) approach, the finite difference time domain (FDTD) method and the multiple-scattering theory (MST) are some of the techniques that have been developed to study the propagation of elastic/acoustic waves in PnCs^34–39^. The transmission-frequency spectrum in the 1D-PnC sensor has been significantly calculated using the TMM.

Our theoretical analysis could also be represented in the form of the different nuclear fuel pellets, the mechanical properties of the materials used in our sensor, the transfer matrix method, and the equations that describe the performance sensor. Therefore, this section is summarized in the form of four subsections:

### Fuel materials

High-burnup nuclear fuels are nuclear fuels that have been subjected to a significant amount of irradiation, leading to modifications in their physical, and chemical characteristics, such as alterations in the O/M ratio and porosity rate, which can have an impact on their thermal conductivity and mechanical properties^11,13^. When evaluating different irradiation behaviors of the fuel pellets, such as mechanical interaction between the pellet and cladding, it is important to consider how porosity, Pu content, and oxygen-to-metal ratio (O/M) affect MOX's mechanical properties^6,40^. The behavior of high-burnup nuclear fuels depends on various factors such as irradiation conditions: As nuclear fuels’ burnup—the quantity of energy they produce per unit mass—increases, their behaviors alter. High burnup exposes the fuel to significant radiation damage, which can alter its mechanical and microstructural characteristics^6^. Temperature: The fuel's temperature has an impact on how it behaves. The fuel may undergo thermal expansion at high temperatures, which may result in stress and deformation. The mechanical characteristics of the fuel, such as its Young modulus, can also be impacted by temperature^41^. Impurities: Impurities in the fuel can also have an impact on how it behaves. For instance, fission products can build up in the fuel and alter its microstructure and mechanical characteristics. Fuel composition: The behavior of fuel can be influenced by its composition^42^. For instance, when comparing traditional uranium oxide fuel with MOX fuel, which contains plutonium, differing properties can be observed.

In brief: the behavior of high-burnup nuclear fuels is complex and depends on some variables, including temperature, impurities, irradiation conditions, and fuel composition^8,39,43^.

#### *CeO*_*2*_*-fuel*

Due to its identical structure, chemical compositions, and mechanical properties, Cerium Oxide, also known as CeO_2_, has been shown as a surrogate material to understand irradiated Mixed Oxide (MOX) based matrix fuel for nuclear power plants^3^. Also, due to its equal fluorite-type structure and comparable microstructural and thermal properties, CeO_2_ has been demonstrated as a surrogate material to understand and simulate irradiated UO_2_-based matrix fuel^3^. For these reasons, almost experimental works used the CeO_2_ as a reference and measured before and after the nuclear fuels studies whether for UO_2_ or (MOX) fuels. The sound speeds in a CeO_2_ pellet were measured, and the consistency of the CeO_2_ values was validated before and after the MOX-pellet investigations^11^. As a result of measurements of several specimens, the data were considered to be stable^3^. For these reasons, this fuel is used as a reference for the other nuclear fuel pellets.

#### *UO*_*2*_*-fuel*

UO_2_ is a material that is used as a fuel in most nuclear reactors, and its elastic characteristics have been a topic of research for many years^15,44^. Their use especially refers to reactor transient and pellet cladding mechanical interaction (PCMI) situations where creep is unlikely to occur^44^. In addition, it is the fuel material used in pressurized water reactors and designed in the shape of pellets, which may or may not also contain plutonium dioxide of 8 mm in diameter. The pellets are stacked on a length of 3.6 m of Zircaloy tubing, which is sealed at both ends with two Zircaloy plugs and pressurized with helium^8^.

#### *PuO*_*2*_*-fuel*

The radioisotope plutonium-238’s thermal output is used as fuel in the thermal to electrical power system on recent deep space missions. The fuel used in these deep space power systems is often in the oxide form, such as plutonium-238 dioxide, because using plutonium in its elemental condition has many disadvantages (^238^PuO_2_). As an oxide, plutonium dioxide is processed into fuel pellets using “traditional” ceramic processing unit procedures like pressing, sintering, and powder sieving. In order to better understand and regulate the processing parameters and further improve the desired properties of the ^238^PuO_2_ fuel pellets, modeling the operations of these units can be helpful^2^.

#### MOX-fuel

Reprocessing UO_2_ spent fuels to recover uranium and plutonium that form during irradiation has been chosen, as a way to protect natural uranium resources and decrease the amount of nuclear waste. Depleted uranium and plutonium are combined as the species UO_2_ and PuO_2_ to create the new UO_2_-PuO_2_ fuels called (Mixed Oxides) known as MOX-fuel. MOX-pellets are manufactured industrially via a dry route method that includes stages including fine powders (grinding, mixing, and sieving of powders, followed by uniaxial pressing of the powder mix into pellets) before the final sintering step^4,45,46^. Many countries use MOX fuels in their light-water nuclear reactors, and they could also be used in fast neutron reactors^4^.

Knowing the physical characteristics of nuclear fuels are crucial for fuel performance analysis. As a result, numerous studies on physical properties have been done^2–6^. One of the more significant physical features is the mechanical and thermal properties, which are crucial for assessing many aspects of fuel performance. Where the ultrasound pulse-echo method was used to determine the sound speeds of longitudinal and transverse waves in MOX-pellets^47^. From the measured data, the mechanical properties were calculated using the literature method^6^. Stoichiometry for the compounds that were created ranged widely from hypo- to hyper-stoichiometry. Because the characteristics of (U, Pu) O_2_ greatly change with porosity, Pu-content, and O/M ratio, it is imperative to understand the data reliance on these factors to develop oxide fuels^13,41,42^.

### Mechanical properties

The elastic properties of pellet materials can be estimated using the relationships provided by the physical acoustics theory linking ultrasonic velocities with elastic properties once the longitudinal and transverse (Shear) wave velocities are known^9^. The ultrasound pulse-echo technique is used to measure the pellets' longitudinal and transverse sound velocities. The mechanical properties of nuclear fuels are shown in Table 1. As mentioned before, these properties depend on porosity, O/M, and Pu-content. Ref.^11^ set a single equation to calculate the velocity for the longitudinal and transverse waves of the MOX-pellet at different porosity, O/M, and Pu-content, as follow:

1 $${C}_{Tj} =5358(1-1.3172P)(1-0.7279x)(1+0.040{c}_{Pu})$$

2 $${C}_{Lj} =2750(1-0.8945P)(1-1.0545x)(1+0.043{c}_{Pu})$$

where $P$ is the porosity of the pellets, x is the deviation of O/M as (U, Pu) O_2–*x*_, and ${c}_{Pu}$ is the Pu-content.

**Table 1:** Different samples of nuclear fuel pellets with their mechanical properties.

Specimens of pellets (samples)	Density (kg/m^3^)	${\mathrm{C}}_{t}$ (m/s)	${\mathrm{C}}_{l}$ (m/s)
CeO_2_	7302	3285	6223
PuO_2_	10,747	2713	5094
UO_2_	10,357	2621.9	4966.5
(U_0.8_Pu_0.2_) O_2_with($\rho =0.143)$	9477	2407	4380

The mechanical properties can be measured from the sound speed (velocity)^11^:

### TMM

To show how waves propagate through space and time, the 1D partial differential equation for wave propagation is mathematically defined as in the following equation^48–50^:

3 $$ \nabla^{2} \phi = c_{L}^{ - 2} \ddot{\phi },\quad {\text{for}}\;{\text{longitudinal}}\;{\text{wave}}, $$

4 $$ \nabla^{2} \phi = c_{T}^{ - 2} \ddot{\phi },\quad {\text{for}}\;{\text{transverse}}\;{\text{wave}}, $$

where $\phi$ is the displacement potential, for the normal incidence case ${v}_{x}=\partial \phi /\partial x$ is the displacement component, $c_{L} = \sqrt {\left( {\lambda + 2\mu } \right)/\rho } \;{\text{and}}\;c_{T} = \sqrt {\mu /\rho }$, and $\nabla^{2} = \partial^{2} /\partial x^{2}$.

The TMM of the periodic structures depends on applying the continuity conditions at interfaces between materials. The harmonic solution of Eqs. (3) and (4) can be expressed as follows by using the separation of variables method^49,51,52^:

For longitudinal wave

5 $${\phi }_{j}({\xi }_{j},t)=[{\alpha }_{1}\mathit{exp}(-i{q}_{Lj}{\xi }_{j})+{\alpha }_{2}\mathit{exp}(i{q}_{Lj}{\xi }_{j})]\mathit{exp}(-i\omega t),$$

For transverse wave

6 $${\phi }_{j}({\xi }_{j},t)=[{\beta }_{1}\mathit{exp}(-i{q}_{Tj}{\xi }_{j})+{\beta }_{2}\mathit{exp}(i{q}_{Tj}{\xi }_{j})]\mathit{exp}(-i\omega t),$$

where ${\alpha }_{1},{\alpha }_{2},{\beta }_{1},{\beta }_{2}$ are unknown coefficients to be determined and, $0 \le \xi_{j} \le \zeta_{j} = a_{j} /\overline{a}_{1} \left( {j = 1,2} \right),\;q_{Lj} = \frac{{\omega \overline{a}_{1} }}{{c_{Lj} }},\;q_{Tj} = \frac{{\omega \overline{a}_{1} }}{{c_{Tj} }}$, $\zeta_{j}$ are the dimensionless thickness of each layer, $\overline{a}_{1}$ is the mean value thickness of the material, i^2^ =  − 1, and ω is the angular frequency.

The dimensionless displacement and stress components can be expressed as^49^:

7 $$ v_{x} = \frac{\partial \phi }{{\partial \xi }},\quad \sigma_{x} = \lambda \left( {\frac{{\partial^{2} \phi }}{{\partial \xi^{2} }}} \right) + 2\mu \left( {\frac{{\partial^{2} \phi }}{{\partial \xi^{2} }}} \right). $$

where $\lambda , \mu$ are lame coefficients that describe the mechanical properties of any solid materials. In the study that follows, we will see how this equation creates the state vectors.

The analytical process is the same for both longitudinal and transverse wave propagation. In the ith unit cell, the boundary conditions of the two layers’ left, and right are stated as^31,39,49,51,53^:

8 $$ v_{xjl}^{\left( i \right)} = \left( {\frac{{\partial \phi^{\left( i \right)} }}{{\partial \xi_{j} }}} \right)\left( 0 \right),v_{xjR}^{\left( i \right)} = \left( {\frac{{\partial \phi^{\left( i \right)} }}{{\partial \xi_{j} }}} \right)\left( {\zeta_{j} } \right), $$

9 $$ \sigma_{xjl}^{\left( i \right)} = \lambda_{j} \left( {\frac{{\partial^{2} \phi^{\left( i \right)} }}{{\partial \xi^{2}_{j} }}} \right)\left( 0 \right) + 2\mu_{j} \left( {\frac{{\partial^{2} \phi^{\left( i \right)} }}{{\partial \xi^{2}_{j} }}} \right)\left( 0 \right), $$

10 $$ \sigma_{xjR}^{\left( i \right)} = \lambda_{j} \left( {\frac{{\partial^{2} \phi^{\left( i \right)} }}{{\partial \xi^{2}_{j} }}} \right)\left( {\zeta_{j} } \right) + 2\mu_{j} \left( {\frac{{\partial^{2} \phi^{\left( i \right)} }}{{\partial \xi^{2}_{j} }}} \right)\left( {\zeta_{j} } \right), $$

The subscripts (*l* and *R*) refer to the left and right sides of the two materials.

By substituting Eqs. (5), (6), and (7) into Eqs. (8), (9), and (10), the following matrix equation can be obtained:

11 $$ V_{jR}^{\left( i \right)} = T_{j}{\prime} V_{jl}^{\left( i \right)} \quad \left( {j = 1,2;\;i = 1,2, \ldots ,n} \right), $$

$V_{jR}^{\left( i \right)} = \left\{ {\sigma_{xjR}^{\left( i \right)} } \right.,\left. {v_{xjR}^{\left( i \right)} } \right\}^{T} ,\quad V_{jl}^{\left( i \right)} = \left\{ {\sigma_{xjl}^{\left( i \right)} } \right.,\left. {v_{xjl}^{\left( i \right)} } \right\}^{T}$ are the state vectors at the right and left sides of the two layers and ${T}_{j}{\prime}$ are 2 × 2 transfer matrix of each layer.

The elements of the transfer matrix (for longitudinal wave) are given by

12 $$ \begin{aligned} T_{j}^{{\prime }} \left( {1,1} \right) & = T_{j}^{{\prime }} \left( {2,2} \right) = \frac{{\left[ {\exp \left( { - iq_{Lj} \zeta_{j} } \right) + \exp \left( {iq_{Lj} \zeta_{j} } \right)} \right]}}{2}, \\ T_{j}^{{\prime }} \left( {1,2} \right) & = \frac{{iq_{Lj} \left( {\lambda_{j} + 2\mu_{j} } \right).\left[ {\exp \left( {iq_{Lj} \zeta_{j} } \right) - \exp \left( { - iq_{Lj} \zeta_{j} } \right)} \right]}}{2}, \\ T_{j}^{{\prime }} \left( {2,1} \right) & = \frac{{i\left[ {\exp \left( { - iq_{Lj} \zeta_{j} } \right) - \exp \left( {iq_{Lj} \zeta_{j} } \right)} \right]}}{{2q_{Lj} \left( {\lambda_{j} + 2\mu_{j} } \right)}}. \\ \end{aligned} $$

Similar analysis can be performed on a transverse wave, and the elements of the transfer matrix are given by:

13 $$ \begin{aligned} T_{j}^{{\prime }} \left( {1,1} \right) & = T_{j}^{{\prime }} \left( {2,2} \right) = \frac{{\left[ {\exp \left( { - iq_{Tj} \zeta_{j} } \right) + \exp \left( {iq_{Tj} \zeta_{j} } \right)} \right]}}{2}, \\ T_{j}^{{\prime }} \left( {1,2} \right) & = \frac{{iq_{Tj} \mu_{j} .\left[ {\exp \left( {iq_{Tj} \zeta_{j} } \right) - \exp \left( { - iq_{Tj} \zeta_{j} } \right)} \right]}}{2},T_{j}{\prime} \left( {2,1} \right) = \frac{{i\left[ {\exp \left( { - iq_{Tj} \zeta_{j} } \right) - \exp \left( {iq_{Tj} \zeta_{j} } \right)} \right]}}{{2q_{Tj} \mu_{j} }}. \\ \end{aligned} $$

At the interface between layers, this condition is satisfied:

14 $${V}_{1R}^{(i)}={V}_{2l}^{(i)}.$$

So, the relationship between the right and left sides of the $i$th unit cell can be obtained from Eq. (11) as

15 $${V}_{2R}^{(i)}={T}_{i}{V}_{1l}^{\left(i\right)}\left(i=\mathrm{1,2},...,n\right),$$

where ${T}_{i}$ is the transfer matrix of the $i$th unit cell and can be written as follows:

16 $${T}_{i}={T}_{2}{\prime}{T}_{1}{\prime}.$$

The TMM is explained in this part in a way that is mostly dependent on the reflection and transmission coefficients.

The reflection and transmission coefficients for the displacement field are given during the propagation of a plane wave in the successive multilayer structure as follow^39,50,51^.

17 $$ \frac{{U_{1} }}{{U_{0} }} = \frac{{T_{12} + E_{0} T_{11} - E_{0} E_{e} T_{21} - E_{e} T_{22} }}{{E_{0} \left( {T_{11} - E_{e} T_{21} } \right) - \left( {T_{12} - E_{e} T_{22} } \right)}},\quad {\text{for}}\;{\text{the}}\;{\text{reflected}}\;{\text{waves}} $$

18 $$ \frac{{U_{e} }}{{U_{0} }} = \frac{{2E_{0} \left( {T_{11} T_{22} - T_{12} T_{21} } \right)}}{{E_{0} \left( {T_{11} - E_{e} T_{21} } \right) - \left( {T_{12} - E_{e} T_{22} } \right)}},\quad {\text{for}}\;{\text{the}}\;{\text{transmitted}}\;{\text{waves}}, $$

where ${E}_{0}$ and ${E}_{e}$ are Young’s moduli of the two semi-infinite solids to the left and right of the PnC structure, and ${U}_{1}$, ${U}_{e},$ and ${U}_{o}$ are the reflected, transmitted, and incident amplitude, respectively. ${T}_{ij}=T(i,j)$ are the elements of the total transfer matrix.

### Sensor performance

The effectiveness and performance of any sensor type can be described by the values of many variables, including the sensitivity (S), the figure of merits (FOM), the quality factor (Q.F), the signal-to-noise ratio (SNR), the detection limit (DL), and the sensor resolution (SR)^19,20,27,49,54^. To obtain these parameters, the following equations can be utilized,

19 $$S=\left(\frac{\Delta {f}_{res}}{{\Delta C}_{t}}\right)$$

20 $$QF=\left(\frac{{f}_{res}}{\Delta {f}_{1/2}}\right)$$

21 $$SNR=\left(\frac{\Delta {f}_{res}}{\Delta {f}_{1/2}}\right)$$

22 $$DL=\left(\frac{{f}_{res}}{20 S (QF)}\right)$$

23 $$SR=\left(DL\right)\left(S\right)$$

24 $$FOM=\left(\frac{S}{\Delta {f}_{1/2}}\right)$$

where ${f}_{res}$ is the resonance frequency of the defect mode, and ${f}_{1/2}$ is the full width at half the maximum of the resonance frequency.

## Numerical results

**Figure 2:**
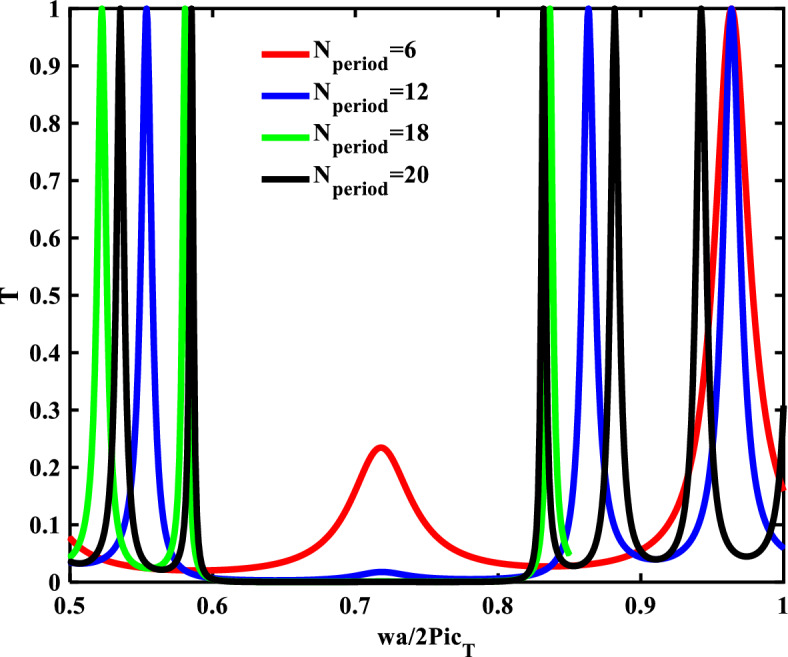
Transmittance spectra of 1D-Binary structure at different N-period.

Due to the interaction of our structure with the incident radiation, the numerical results are focused on the transmittance spectra of our design. The numerical findings are essentially based on the TMM and the experimental fitting that is completely explained in the previous. In order to simulate the radiation's interaction with our suggested design, the Matlab codes would be prepared. The considered structure consists of two materials Si and SiO_2_, such that their mechanical and geometrical parameters of these materials are represented in Table 2. Figure 2, describes the transmittance spectra of the binary1D-PnC at different numbers of periods that are configured as (Si/SiO_2_)^N^ in the normal incident case. Equation (18) provides a formula for the coefficient that expresses the percentage of waves that are transmitted through the structure. This figure aims to optimize our structure and get the optimum phononic band gap (PnBG) with the highest width and intensity. With increasing the number of periods from 6 to 20, the change in the transmittance spectra was observed, and a significant influence in the PnBG is observed. The PnBG becomes sharper and steeper at N = 18 and N = 20. The PnBG resulted from the constructive interference between the incident and reflected elastic waves at the interfaces of the constituent materials. Due to the differences in their constituent mechanical properties (density, sound speed, and thickness), the elastic wave that incident on the surface of PnCs splits into several waves. The diffracted waves then interfere with one another at each interface. The creation of the PnBG occurs if the interference was constructive^34,48^. The interfaces between the periodic materials are formed as the periodicity number inside the PnC structure increases. As a result, the band gaps become clearer as the periodicity number N increases. To make the PnBG more appropriate for our calculation, N = 18 was chosen in the following calculation.

**Table 2:** The values of the geometrical parameters and the mechanical properties of the materials used in the calculation.

Material	Density (kg/m^3^)	${\mathrm{C}}_{t}$ (m/s)	${\mathrm{C}}_{l}$ (m/s)	Thickness $(\mathrm{\mu m})$
Silicon (Si)	2330	5843	8433	2
Silicon dioxide (SiO_2_)	2648	3263	5175	1

**Figure 3:**
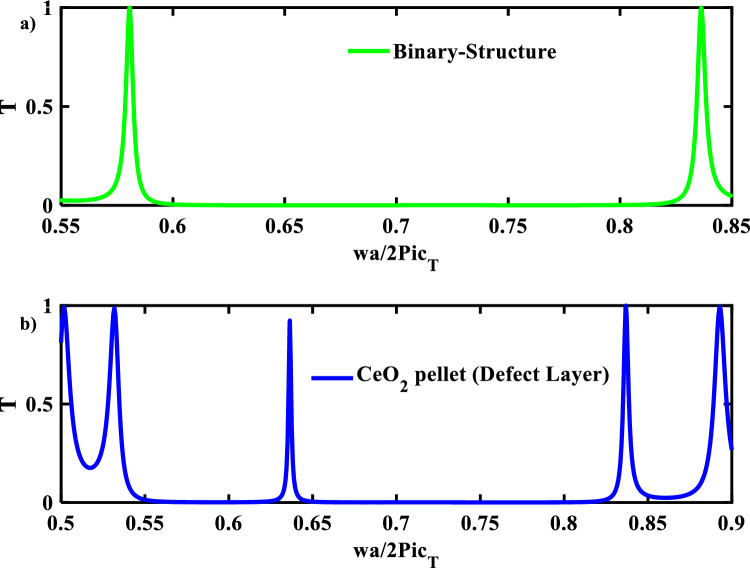
Transmittance spectra of 1D PnC (**a**) Binary structure and (**b**) defective structure with CeO_2_-Pellet as a defect layer.

For this structure to act as a sensor for the irradiation behaviors of nuclear fuel pellets in reactors, a defect layer (material) is introduced through the designed structure such that, (Si/SiO_2_)^9^(D-layer) (Si/SiO_2_)^9^. The creation of resonant mode within the band gaps is a result of incident elastic wave energy that is stored partially by the defect layer. The resonant mode frequency and intensity will change if any physical property of the defect layer is changed. Therefore, the type and physical characteristics of the defect layer (nuclear fuels) can be detected by PnCs. Notably, the mechanical properties of each nuclear fuel differ from one another. The defect layer is firstly filled with CeO_2_-fuel as a baseline for other nuclear fuels because almost experimental works used the CeO_2_-fuel as a reference. The mechanical properties of the CeO_2_-fuel are listed in Table 1 and the optimum thickness of the defect layer is ${d}_{d}=1.8 \mathrm{\mu m}$. Figure 3, shows the transmittance spectra versus the normalized frequency $\left(\omega a/2\pi {c}_{t}\right)$ with and without a defect layer in our structure. The resonance mode appeared inside the PnBG of our structure. In particular, the symmetry and periodicity of the PnC structure are broken when a defect layer is added inside it^49^. The output results based on the mentioned equations were introduced in the theoretical part section. As shown in Fig. 3, the width of the PnBG in the case of the defect structure is greater than that of the binary structure and appears at the normalized frequency range between (0.534–0.835). In addition, the defect mode appears at a resonance frequency equal to (${f}_{res}=692.157\times {10}^{6}\mathrm{Hz})$ with intensity equal (92.481%).

**Figure 4:**
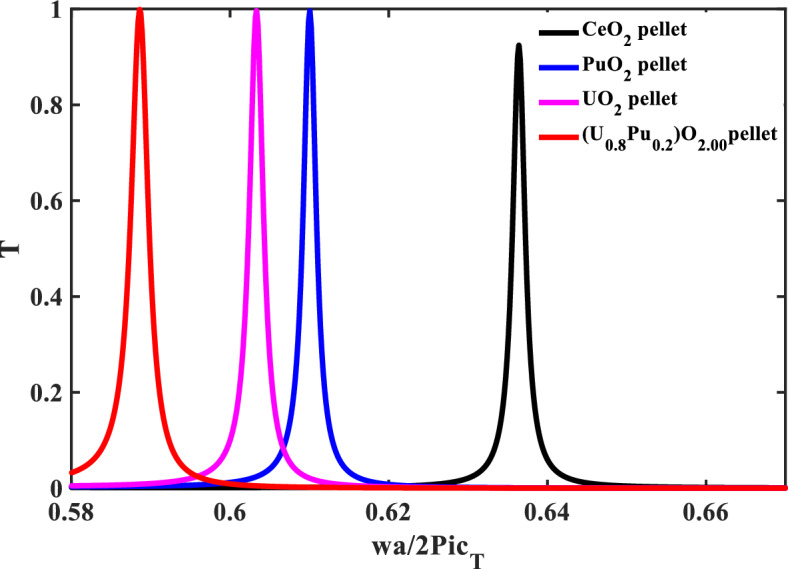
Transmittance spectra of the proposed sensor with different defective layers from pellets used in nuclear reactors.

**Figure 5:**
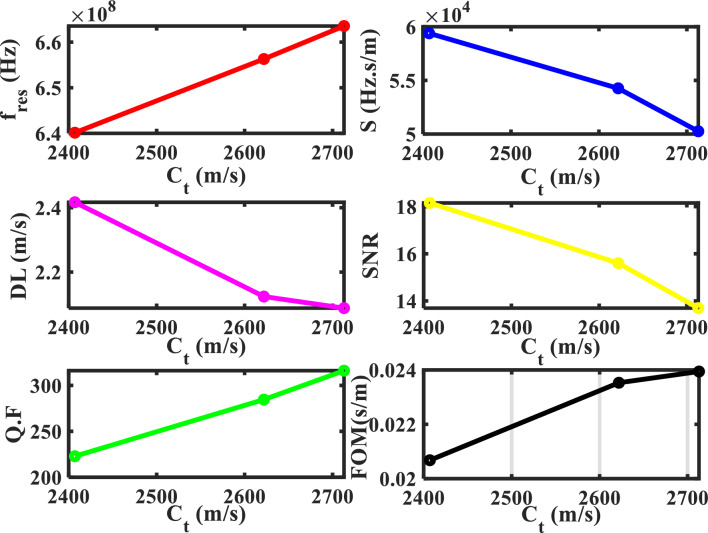
The performance parameters for each pellet used as a defect layer in the 1D-DPnC.

In the following, the defect layer will be filled with different types of nuclear fuels that will be introduced separately. In addition, the resonance frequency of the CeO_2_-fuel can be used as a reference for the resonance frequency of the different nuclear fuels-pellets. Figure 4 shows the transmittance spectra with different defective layers from the fuel-pellets used in the nuclear reactors. When the defect layer is filled with different types of nuclear fuels (CeO_2_, UO_2_, PuO_2_, and MOX), the position and intensity of the resonant mode change. The mechanical properties of these nuclear fuels represent the main reason for the change in the transmittance spectra and the effect on the position of the resonant mode. Table 3 clarifies the position of the resonance frequency of each nuclear fuel-pellet. According to this figure, the structure is considered a sensor for different types of nuclear fuel-pellets. Thus, the effectiveness and the performance of this sensor must be shown by calculating the following factors [the sensitivity (S), the figure of merits (FOM), the quality factor (QF), the signal-to-noise ratio (SNR), the detection limit (DL), and the sensor resolution (SR)]. These parameters are shown in Fig. 5 and calculated by Eqs. (19–24). Figure 5, shows the performance parameters at each pellet used as a defect versus the transverse velocity for each pellet. The sensitivity for each nuclear fuel-pellet was calculated and noted that the sensitivity of the MOX-fuel is the highest one with 59.388 × 10^3^ (Hz s/m). This result agrees with the experimental research^11^. Notably, the MOX-fuel had a greater Young's modulus than UO_2_^11^. This parameter can be calculated by knowing the value of the longitudinal and transverse velocity as the following equation^11^:

25 $$ E_{j} = \rho_{j} C_{Tj} { }^{2} \left[ {\left( {3C_{Lj} { }^{2} - 4C_{Tj} { }^{2} } \right)/\left( {C_{Lj} { }^{2} - C_{Tj} { }^{2} } \right)} \right], $$

**Table 3:** The performance parameters at each pellet used as a defect Layer.

Pellets	${\mathrm{C}}_{t}$(m/s)	${\Delta \mathrm{C}}_{t}$(m/s)	f_res_ (Hz) × 10^6^	$\Delta$ f_res_ (Hz) × 10^6^	$\Delta$ f_1/2_ (Hz) × 10^6^	*S* (Hz. s/m) × 10^3^	*Q. F*	*SNR*	*DL* (m/s)	*SR* (Hz) × 10^3^	*FOM* (s/m) × 10^–3^
CeO_2_	3285	–	692.278	–	2.089	–	331.392	–	–	–	–
PuO_2_	2713	572	663.531	28.747	2.099	50.256	316.117	13.695	2.088	104.934	23.942
UO_2_	2621.9	663.1	656.298	35.98	2.306	54.260	284.604	15.602	2.124	115.248	23.529
(U_0.8_Pu_0.2_) O_2_	2407	878	640.135	52.143	2.872	59.388	222.888	18.155	2.417	143.600	20.678

Therefore, the following study will be focused on (MOX) fuel because of its many advantages that have been proven in practice compared to the rest of the nuclear fuels and the theoretical results that have been reached.

The mechanical properties of the (MOX) fuel are dependent on the porosity, the oxygen-to-metal ratio (O/M), and the plutonium (Pu-content). Where, the experimental study^11^ set equations to calculate the velocity for the longitudinal and transverse waves of the MOX-pellet at different porosity, O/M, and Pu-content as shown in Eqs. (1, 2). In what follows, the effect of each factor will be studied on the mechanical properties and the resonance frequency of the (MOX) fuel and how these factors have affected the effectiveness and the performance of this sensor.

### Effect of porosity on the sensor performance

**Figure 6:**
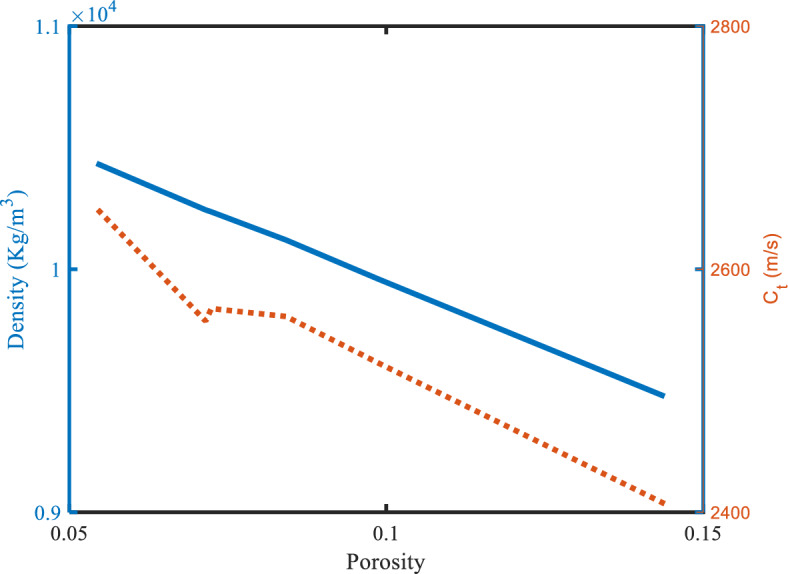
The density and the sound speed of transverse waves of the specimen (U_0.8_ Pu_0.2_) O_2.0_ versus porosity with 20% Pu and 2.00 O/M.

When the defect layer is filled with specimens of (U_0.8_ Pu_0.2_) O_2.0_ (MOX-fuel) with different values of porosity, the resonance frequency of the defect mode changes. In particular, these values influence the mechanical properties of the MOX fuel and then influence the transmittance spectra. The mechanical properties (density and transverse velocity) of the MOX-fuel versus the porosity of this fuel are plotted in Fig. 6. As shown in Fig. 6, the increase in porosity decreases the velocity and the density of the MOX-fuel. This agrees with the measured data and equation present in Ref.^11^. Where the fitted liner equation of the transverse velocity of this Ref. is as follows:

26 $$ C_{{T\left( {MOX} \right)}} { } = 2774\left( {1 - 0.8945P} \right),\quad {\text{with}}\;\left( {{\text{Pu}} = {2}0\% ,\;{\text{O}}/{\text{M}} = {2}.00} \right) $$

**Figure 7:**
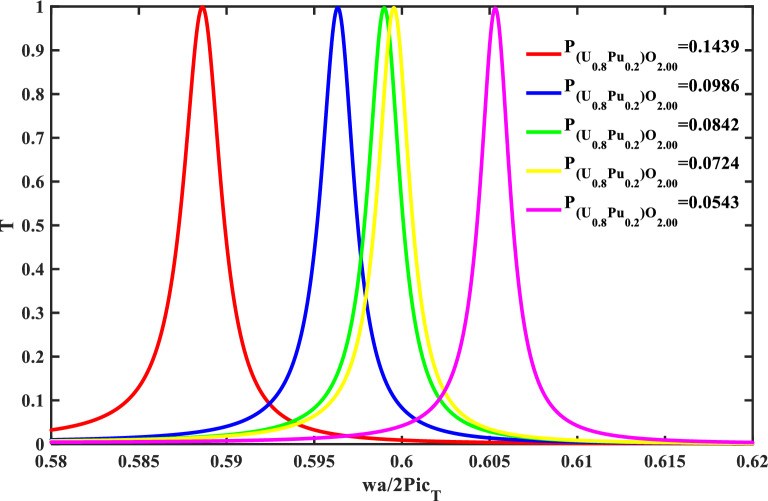
The effect of porosity of specimen (U_0.8_ Pu_0.2_)O_2.00_ with 20% Pu and 2.00 O/M on the resonance wavelength of the transmittance spectrum of defective structure.

**Figure 8:**
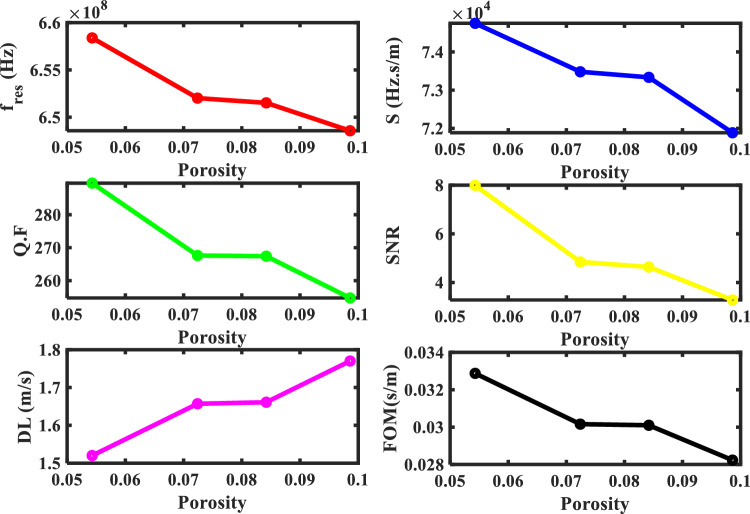
The performance parameters at different porosity of the MOX-pellet with used as a defect layer.

This equation can be substituted into the following equation ${C}_{Tj} =\sqrt{{\mu }_{j}/{\rho }_{j}}$, where ${\mu }_{j}$ is the lame coefficient that describes the mechanical properties of any solid materials and measures the density of this nuclear fuel. For the fitted linear equation of our study according to Fig. 6, the transverse velocity, and the density as a function of porosity are shown the follows:

27 $$ C_{{T\left( {MOX} \right)}} { } = - 2469.2P + 2760.4,\quad {\text{with}}\;\left( {{\text{Pu}} = {2}0\% ,\;{\text{O}}/{\text{M}} = {2}.00} \right) $$

28 $$ \rho_{{\left( {MOX} \right)}} { } = - 10658P + 11011,\quad {\text{with}}\;\left( {{\text{Pu}} = {2}0\% ,\;{\text{O}}/{\text{M}} = {2}.00} \right) $$

where R^2^ = 9.32 is the normal residual, for this reason, these equations agree with the experimental result. The values of the transverse velocity at different porosity are present in Table 4. The negative correlation between the porosity and the velocity will be reflected in the other mechanical properties and the resonance frequency of the defect mode. As shown in Fig. 7, the resonance frequency changed from 640.222 × 10^6^ to 658.386 × 10^6^ Hz when the MOX-fuel was used with different porosity from (0.1439 to 0.0543). The performance parameters of our structure are calculated at different values of porosity and are shown in Table 4 and Fig. 8. Where the specimen of the MOX-fuel with the lowest porosity (0.0543) has the highest sensitivity compared to the other values and equal 74.748 (Hz s/m).

**Table 4:** The performance parameters at different values of the porosity of the MOX-fuel used as a defect.

The porosity of (U_0.8_Pu_0.2_) O_2_	${\mathrm{C}}_{t}$(m/s)	${\Delta \mathrm{C}}_{t}$(m/s)	f_res_ (Hz) × 10^6^	$\Delta$ f_res_ (Hz) × 10^6^	$\Delta$ f_1/2_ (Hz) × 10^6^	*S* (Hz s/m) × 10^3^	*Q. F*	*SNR*	*DL* (m/s)	*SR* (Hz) × 10^3^	*FOM* (s/m) × 10^−3^
0.1439	2407.0	–	640.222	–	2.85	–	224.639	–	–	–	–
0.0986	2523.2	116.2	648.575	8.353	2.546	71.8846	254.742	3.2808	1.770	127.235	28.234
0.0842	2561.1	154.1	651.523	11.301	2.4364	73.335	267.412	4.638	1.661	121.809	30.099
0.0724	2567.6	160.6	652.023	11.801	2.4364	73.4806	267.617	4.843	1.657	121.757	30.159
0.0543	2650.0	243	658.386	18.164	2.2736	74.7489	289.578	7.989	1.520	113.618	32.876

### Effect of O/M on the sensor performance

**Figure 9:**
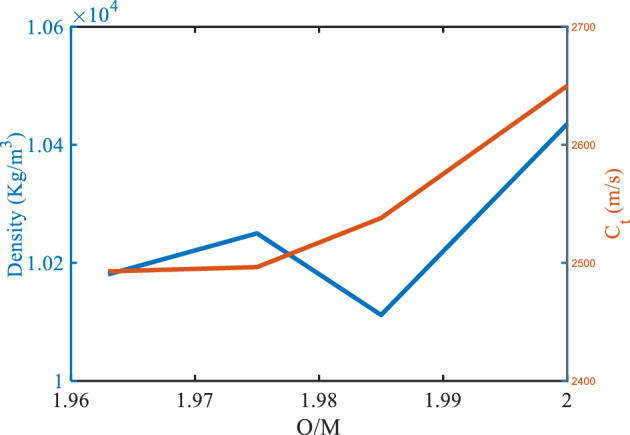
The density and the sound speed of transverse waves of the MOX-pellets with 20% Pu and zero porosity as a function of O/M.

**Figure 10:**
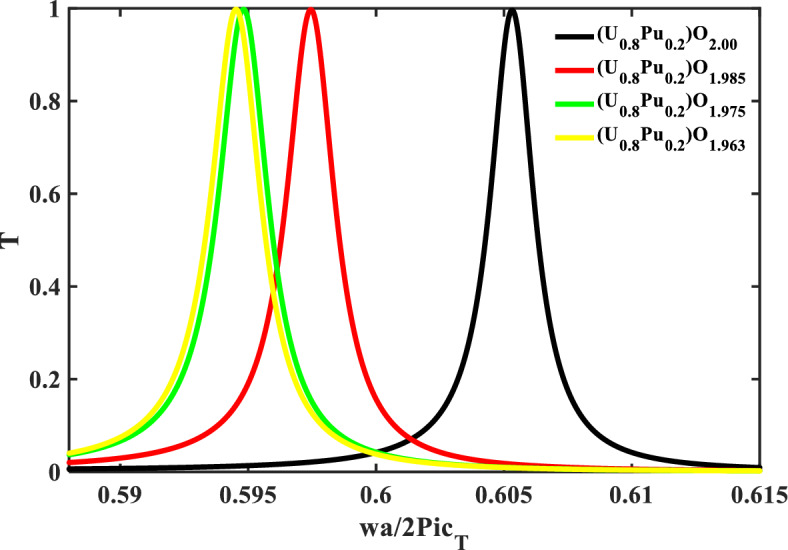
The effect of O/M of the MOX-pellets with 20% Pu and the porosity-free value on the resonance wavelength of the transmittance spectrum of defective structure.

To investigate the impact of O/M or Pu-content on the mechanical properties and transmittance spectra, the data that including with a different O/M and Pu-contents might be assumed with a 0% porosity (the porosity-free values). The effect of the O/M on the resonance frequency of the defect layer is shown in Fig. 10 with ($\rho =0$, and Pu = 20%). Where the defect layer is filled with different specimens of (U_0.8_ Pu_0.2_) O_2−x_ and x is the deviation from the stoichiometry of (U_0.8_ Pu_0.2_) O_2−x_. As mentioned before, if the percentage of O/M was changed in the specimen, this leads to a change in the mechanical properties of the specimen, and thus a change in the position of the resonance frequency in the transmittance spectra. Therefore, the mechanical properties (density and transverse velocity) of the MOX-fuel were shown versus the O/M of this fuel in Fig. 9. As shown, the O/M decreases the density of the MOX-fuel. Also, Fig. 9, shows that the transverse velocity decreases in the hypo-stoichiometric region and this agrees with the measured data and equation present in Ref.^11^,

29 $${C}_{T(\rho\; = \;0,\;\mathrm{ and \;Pu}\;= \;20\mathrm{\%}) }=2774\left(1-1.0545x\right).$$

**Figure 11:**
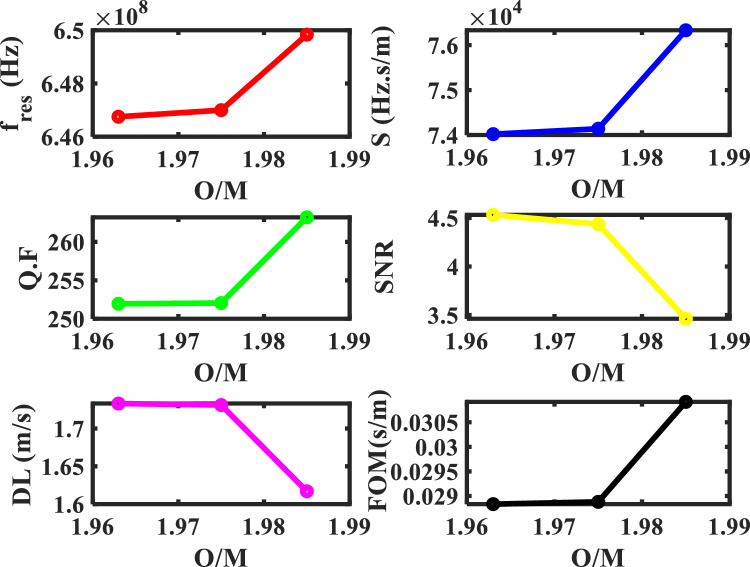
The performance parameters of our structure as a function of O/M of the MOX-pellet.

Figure 10, shows the effect of O/M on the resonance frequency with ($\rho =0$, and Pu = 20%). The position of the resonance frequency shifts to a lower frequency with an increase in the deviation of the stoichiometry of (U_0.8_ Pu_0.2_) O_2–x_, and also this agrees with the previous figure. After that, the performance was shown of our structure at the range of prepared O/M (1.963–2.000) as shown in Table 5 and Fig. 11.

**Table 5:** The performance parameters at different values of O/M of the MOX-fuel used as a defect layer.

O/M of the MOX-pellet	${\mathrm{C}}_{t}$(m/s)	${\Delta \mathrm{C}}_{t}$(m/s)	f_res_ (Hz) × 10^6^	$\Delta$ f_res_ (Hz) × 10^6^	$\Delta$ f_1/2_ (Hz) × 10^6^	*S* (Hz s/m) × 10^3^	*Q. F*	*SNR*	*DL* (m/s)	*SR* (Hz) × 10^3^	*FOM* (s/m) × 10^−3^
(U_0.8_Pu_0.2_) O_2_	2650.0	–	658.386	–	2.240	–	–	–	–	–	–
(U_0.8_Pu_0.2_) O_1.985_	2538.0	112	649.837	8.549	2.469	76.330	263.198	3.462	1.617	123.425	30.915
(U_0.8_Pu_0.2_) O_1.975_	2496.4	153.6	646.998	11.388	2.567	74.140	252.044	4.436	1.731	128.336	28.881
(U_0.8_Pu_0.2_) O_1.963_	2492.8	157.2	646.750	11.636	2.567	74.020	251.947	4.532	1.733	128.350	28.835

### Effect of Pu-content on the sensor performance

**Figure 12:**
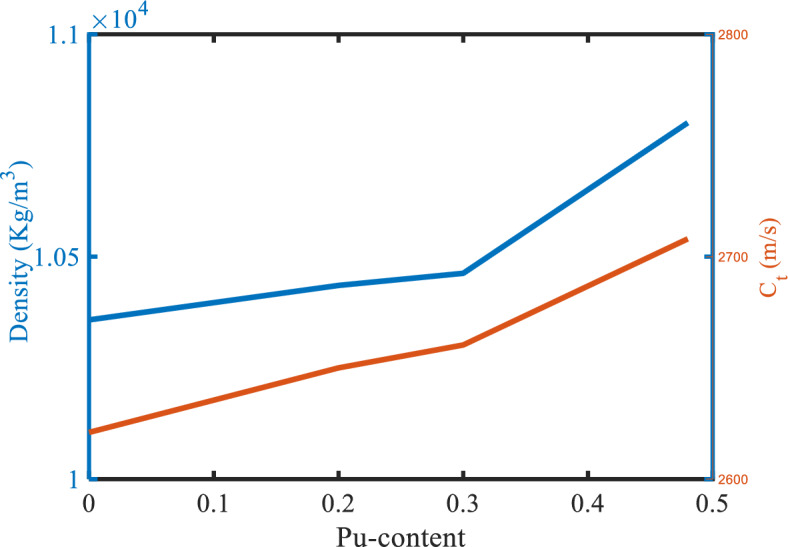
The density and the sound speed of transverse waves of the MOX-pellets with 2.00 O/M and the porosity-free value as a function of Pu-content.

**Figure 13:**
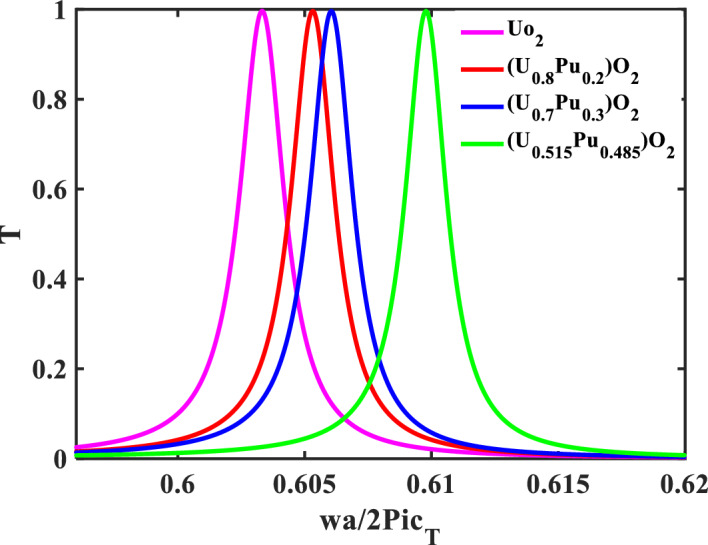
The effect of Pu-content of the MOX-pellets with 2.00 O/M and the porosity-free value on the resonance wavelength of the transmittance spectrum of defective structure.

**Figure 14:**
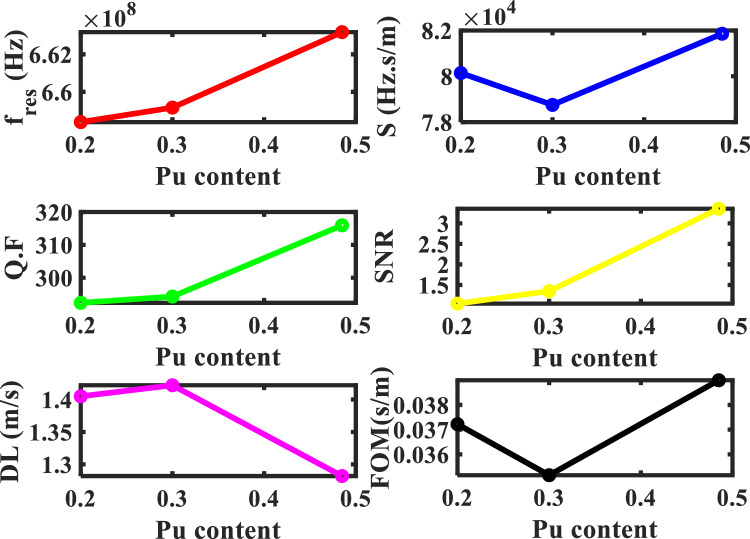
The performance parameters of our structure as a function of Pu-content of the MOX-pellet.

The impact of Pu-content on the mechanical properties and transmittance spectra with 2.00 O/M and $\rho =0$ is shown in Figs. 12, and 13. Where the defect layer is filled with different specimens of Pu-contents from (U_0.8_ Pu_0.2_) O_2_. Also, if the percentage of Pu-content was changed in the specimen, this leads to a change in the mechanical properties of the specimen, and thus a change in the position of the resonance frequency in the transmittance spectra. The mechanical properties (density and transverse velocity) of the MOX-fuel versus the Pu-content of this fuel are in Fig. 12. As shown in Fig. 12, the transverse velocity is higher for the specimens with higher Pu-content. In the case of zero Pu-content, this means that the specimen is UO_2_ with transverse velocity (${\mathrm{C}}_{t}=2621.9\mathrm{ m}/\mathrm{s})$. With an increase in the percentage of the Pu-content, the transverse velocity increases. The behavior of the density is based on the behavior of the transverse velocity. Figure 13, shows the effect of Pu-content which is present in the specimen on the resonance frequency of the defect mode. With increasing the percentage of the Pu-content, the resonance frequency shifts to a higher frequency value. This reflects positively on the performance of the sensor which represents in Table 6 and Fig. 14. After this study, we can conclude that the effect of porosity is remarkable compared to the percentage of O/M, and Pu-content.

**Table 6:** The performance parameters at different values of the Pu-content of the MOX-fuel each pellet used as a defect layer.

Pu-content of the MOX-pellet	${\mathrm{C}}_{\mathrm{t}}$(m/s)	${\Delta \mathrm{C}}_{\mathrm{t}}$(m/s)	f_res_ (Hz) × 10^6^	$\Delta$ f_res_ (Hz) × 10^6^	$\Delta$ f_1/2_ (Hz) × 10^6^	S (Hz. s/m) × 10^3^	Q. F	SNR	DL (m/s)	SR (Hz) × 10^3^	FOM (s/m) × 10^−3^
UO_2_	2621.9	–	656.134	–	2.306	–	284.533	–	–	–	–
(U_0.8_Pu_0.2_) O_2_	2650	28.1	658.386	2.252	2.153	80.142	292.356	1.045	1.405	112.600	37.22
(U_0.7_Pu_0.3_) O_2_	2660.3	38.4	659.158	3.024	2.24	78.75	294.266	1.35	1.4	111.982	35.156
(U_0.515_Pu_0.485_) O_2_	2708	86.1	663.182	7.048	2.099	81.858	315.951	3.357	1.282	104.941	38.998

### Fabrication facilities and experimental verification

#### Nuclear verification

For the specimen preparation, the researchers utilized UO_2_, PuO_2_, and MOX powders with varying Pu concentrations to fabricate MOX pellets with different levels of Pu (ranging from 0% to 100%). These powders were adjusted in nitrate solution and converted into powders using microwave heating^55^. Celphere was added to the powders to control density and increase the porosity of the sintered pellets. The powders were then compressed into green pellets with dimensions of approximately 5 mm diameter and 7 mm length. The pellets were subjected to dewaxing at 1073 K and sintered at 1923 K in an Ar/H_2_ mixed gas environment with moisture to regulate the density and porosity of the pellets^43^. After sintering, the pellets were heat-treated to modify the O/M (oxygen-to-metal) ratio to the desired levels based on the oxygen potential of (U,Pu)O_2_^56^. The sintered pellets underwent grinding using an agate mortar for the purpose of X-ray diffraction analysis. The results of the analysis indicated that the specimens possessed a single-phase with a face-centered cubic (FCC) structure that was homogeneous in nature. Furthermore, the lattice parameters of the specimens aligned with Vegard’s law and demonstrated consistency in both Pu content and O/M^42^.

#### Measurements

The ultrasound pulse-echo method was utilized to determine the sound speeds of longitudinal and transverse waves present in MOX pellets. The method of ultrasound pulse-echo was implemented via the utilization of an ultrasonic flaw detector (KJTD Co. Ltd, HIS-3) at a frequency of 5 MHz, and a temperature of 300 K. In order to attain more precise measurement, five data points were taken at five distinct points at the end faces of the pellet and then averaged. The accuracy of the data was ensured by measuring the sound speeds in a CeO_2_ pellet before and after the MOX pellet studies and verifying the consistency of the CeO_2_ values. This process confirmed that the data which was acquired from the MOX pellets was dependable and stable, as evidenced by the measurements of various specimens. The mechanical characteristics were assessed through the use of the sound velocity, and it was determined that the primary factor contributing to the reduction of Young's modulus was porosity^11^.

#### Phononic crystal verification

The experimental validation of PnC structures as micro-periodic multilayers has drawn much attention over the past few decades. Due to their comparatively large sizes, these designs are notable for being more flexible and simpler to manufacture than their equivalents in traditional photonic crystals and photonic crystal fibers. Because of the wide diversity of microproducts, microelectromechanical systems have also received a lot of interest^57^. This is because numerous techniques and methods, including photopolymerization, focused ion beam direct writing, laser chemical vapor deposition, material jetting, powder bed fusion, photolithography, micro stereolithography printing, and filament extrusion, have been used to demonstrate the experimental verification of two and three-dimensional PnC designs^58,59^. But the creation of two- and three-dimensional models PnC structures are complicated and have a variety of challenges, including high costs, difficult designs, mechanical stability, fabrication precision, and surface roughness.

As a result, significant number of researchers have shifted their attention to developing and constructing 1D-DPnCs, owing to their uncomplicated nature, economical production, and exceptional mechanical robustness^60,61^. The photo-lithographic method is widely recognized as a highly favored technique in the construction of 1D-DPnCs as it provides a straightforward and precise approach to fabrication^23^. This technique involves the application of PnC constituent materials onto a photo-resistant layer, which can then be coated on a substrate composed of materials such as lead or epoxy, by utilizing an optical mask. The process of reactive ion etching can be employed to deposit the constituent materials of the PnC structure onto the substrate, thereby enabling the formation of a grooved square wave pattern^62^. The investigation of acoustic wave propagation within the fabricated design can be accomplished via the utilization of two transducers. The initial transducer is linked to both a power amplifier and signal generator, which serve to generate the incident acoustic waves. The secondary transducer, on the other hand, is responsible for the recording of the structure's transmissivity or reflectivity and is typically passed over an oscilloscope or computing system^61,63^.

### Ethics approval

This article does not contain any studies involving animals or human participants performed by any of the authors.

## Conclusions

To sum up, we reported in this article a novel application of a 1D-PnC sensor for monitoring and simulating the irradiation behaviors of the fuel pellets in reactors. These results are expected to help enhance the characterization of nuclear fuel materials. The irradiation behaviors have appeared through the mechanical interactions of the fuel-pellets. The methodology of this study is constructed based on fitting the experimental equations that describe the mechanical properties of these materials, the TMM for computing the transmission spectra, and the sensor performance parameters. The numerical results are summarized in more than one point. The first point, the sensor materials are taken after an optimization strategy to get the optimum parameters. In the second point, different nuclear fuel materials are compared with each other by calculating different performance parameters. In the third point, the MOX-fuel is taken as the target fuel, and we have studied the effects of different parameters such as the porosity, O/M, and Pu-content on its mechanical properties and resonance modes that appeared in the PBG. In addition, the performance parameters were calculated for each parameter. It was concluded that, when the porosity is equal to 0.0543, the sensitivity achieved the highest value of 74.7489 × 10^3^ (Hz s/m). In addition, when the specimen is proposed with the ratio of O/M ((U_0.8_Pu_0.2_) O_1.985_), the highest sensitivity value of 76.330 × 10^3^ (Hz s/m) is obtained. Moreover, when the Pu-content in the specimen is taken as (U_0.8_Pu_0.2_) O_2_, a promising sensitivity with the value of 80.142 × 10^3^ (Hz s/m) is attained by the sensor. Finally, our sensor could help to establish the operating safety margin for nuclear fuel throughout its life in the reactor.

## Data Availability

Requests for materials should be addressed to Arafa H.Aly.
